# A Simple, Inexpensive Device for Nucleic Acid Amplification without Electricity—Toward Instrument-Free Molecular Diagnostics in Low-Resource Settings

**DOI:** 10.1371/journal.pone.0019738

**Published:** 2011-05-09

**Authors:** Paul LaBarre, Kenneth R. Hawkins, Jay Gerlach, Jared Wilmoth, Andrew Beddoe, Jered Singleton, David Boyle, Bernhard Weigl

**Affiliations:** PATH, Seattle, Washington, United States of America; Duke-National University of Singapore Graduate Medical School, Singapore

## Abstract

**Background:**

Molecular assays targeted to nucleic acid (NA) markers are becoming increasingly important to medical diagnostics. However, these are typically confined to wealthy, developed countries; or, to the national reference laboratories of developing-world countries. There are many infectious diseases that are endemic in low-resource settings (LRS) where the lack of simple, instrument-free, NA diagnostic tests is a critical barrier to timely treatment. One of the primary barriers to the practicality and availability of NA assays in LRS has been the complexity and power requirements of polymerase chain reaction (PCR) instrumentation (another is sample preparation).

**Methodology/Principal Findings:**

In this article, we investigate the hypothesis that an electricity-free heater based on exothermic chemical reactions and engineered phase change materials can successfully incubate isothermal NA amplification assays. We assess the heater's equivalence to commercially available PCR instruments through the characterization of the temperature profiles produced, and a minimal method comparison. Versions of the prototype for several different isothermal techniques are presented.

**Conclusions/Significance:**

We demonstrate that an electricity-free heater based on exothermic chemical reactions and engineered phase change materials can successfully incubate isothermal NA amplification assays, and that the results of those assays are not significantly different from ones incubated in parallel in commercially available PCR instruments. These results clearly suggest the potential of the non-instrumented nucleic acid amplification (NINA) heater for molecular diagnostics in LRS. When combined with other innovations in development that eliminate power requirements for sample preparation, cold reagent storage, and readout, the NINA heater will comprise part of a kit that should enable electricity-free NA testing for many important analytes.

## Introduction

Clinical diagnostic assays targeted to nucleic acid (NA) markers are becoming an increasingly important part of the clinician's toolbox. Many disease states are difficult to diagnose due to the lack of specific and well-characterized biomarkers in an accessible specimen. These generalizations apply in particular to infectious disease diagnostics. The clinical signs of infection are often non-specific (e.g., inflammation or fever) and may originate from many possible sources, yet the treatments are more often specific and require an accurate diagnosis to be effective. There are many infectious diseases endemic in LRS where the lack of simple, instrument-free, NA diagnostic tests is a critical barrier to effective treatment, in part because of co-morbidities that confound a differential diagnosis. These diseases include malaria, human immunodeficiency virus (HIV-1), tuberculosis (TB), influenza, and many others.[1] Millions of lives are lost and a huge morbidity burden incurred through inadequate diagnosis and treatment of these diseases.[1] In many cases the need for rapid diagnostics appropriate for these LRS is so severe that mediocre performance tests such as RDT are preferred to less accessible but better performing NA tests.[2] Clearly, any technology that can increase the practicality and availability of NA assays in LRS could have a significant impact on global public health.

Nucleic acid detection, to date, has mainly been confined to wealthy, developed countries or to the large centralized facilities in the developing world that can marshal the resources required to perform these techniques. Like many molecular diagnostic assays, nucleic acid amplification techniques (NAATs) typically require a significant investment in equipment, training, and infrastructure. Economic and infrastructural realities dictate that diagnostics for the developing world need to be foremost inexpensive; but also, accurate, reliable, rugged, and suited to the contexts of these low-resource settings (LRS).[3]–[5] Recent guidelines published by the World Health Organization recommend that diagnostic devices for developing countries should be ASSURED: Affordable, Sensitive, Specific, User-friendly, Rapid and robust, Equipment-free, and Deliverable to end users.[6] In some diagnostic contexts in LRS, rapid diagnostic tests (RDT) based on the immunochromatography strip (ICS) fit the ASSURED model, albeit with limited sensitivity and specificity.[7]–[9] NAAT assays that use polymerase chain reaction (PCR) amplification are capable of providing excellent sensitivity and specificity but generally fail to meet the ASSURED guidelines for affordability, rapidity and robustness, equipment-free operation, and deliverability.[10], [11] Appropriate, low-cost, equipment-free, pathogen-specific NA marker assays that characterize medical care in much of the developing world remain unavailable in LRS.

One of the primary barriers to the practicality and availability of NA assays in LRS has been the complexity of PCR amplification. PCR is inherently impractical in LRS where reliable electrical power, complex equipment, training, reagent storage, quality programs and clean water, are intermittent or absent. [1], [12] Recently, there have been significant developments in a class of NAATs that do not require temperature cycling.[13]–[16] A comprehensive review of these techniques, and their application in LRS has recently been published. [17] These isothermal amplification techniques vary in amplification temperature and duration, as well as complexity of reagents required—and many are proprietary—but all have the potential to be simpler and require less complex equipment than PCR-based assays. These methods use a variety of reaction principles to specifically amplify NA targets through isothermal melting, exponential amplification and intermediate target generation; and which, in several cases, can be detected directly without the need for an instrument.[17]–[20] Nevertheless, almost all investigators and manufacturers currently use some type of electrically powered equipment to achieve and maintain the temperature required for amplification, although this equipment can be much simpler than the typical PCR thermocycler. This inherent simplicity makes isothermal amplification more appropriate for diagnostics in LRS.

One of the letters in the acronym ASSURED — the guideline for providing diagnostics to LRS — represents “equipment free.” We are currently developing a non-instrumented nucleic acid (NINA) platform that requires no detection instrument, no electrical power, no batteries, and no external reagents. We believe this can be achieved by combining isothermal amplification with a novel method for generating the required temperature profile without electrical power in a simple disposable that contains the lyophilized assay reagents. Our first prototype of this platform uses loop-mediated amplification (LAMP) [21] as the model for an isothermal amplification technique and malaria as a model diagnostic target. The amplification protocol requires incubating the reaction mixture at ∼65°C for at least 60 minutes. This temperature requirement is sufficiently flexible that small excursions (+/−1.5°C) around this target are tolerable. [22]–[26] LAMP (and several other isothermal techniques) have been shown to far less sensitive to inhibitors than PCR, to the point where direct assay of whole blood and other unpurified specimens is feasible.[18], [19] In those cases, no power or instruments are required for NA purification, as is the case with PCR. In addition, recent advances in protein stabilization make it likely that the reagents can be dried-down in the reaction tubes with sufficient stability to avoid the need for a cold-chain during delivery and storage. Thus, another power consuming “instrument” is eliminated. We have not yet attempted to package all of these features and advances into a single prototype device; however, the successful demonstration of electricity-free temperature-controlled heating in a disposable format reported here is an important first step toward the long-term goal.

The prototype NINA platform exploits exothermic chemical heating, as used in “ready-to-eat” meals and camping hand warmers. Table S1 summarizes the prior history of prototype development. Hatano and coworkers recently described a crude heater that was able to perform a qualitative LAMP assay for anthrax using off-the shelf pocket hand warmers and a Styrofoam box. [27] Dominguez et al. used a similar container with an unspecified phase change material to maintain a stable incubation temperature for a commercial interferon gamma release assay at 37C (although the heat source was conventional). [28] While these interesting approaches are compelling in their simplicity, the bulky apparatus displayed slow warm-up (>30 min.); and for LAMP, significant temperature variation within incubation time, and a lack of run-to-run repeatability was observed. To meet the performance goals implied in the ASSURED guidelines, an optimized heating unit should be engineered to eliminate or minimize all sources of variation. When combined with the temperature-moderating characteristics of engineered phase change materials (EPCM), we demonstrate that an engineered exothermal chemical heating unit can produce a consistent constant-temperature incubator for isothermal NA amplification suitable for a variety of isothermal techniques.

## Results and Discussion

### Heat Production and Temperature in the NINA Heater

**Figure 1:**
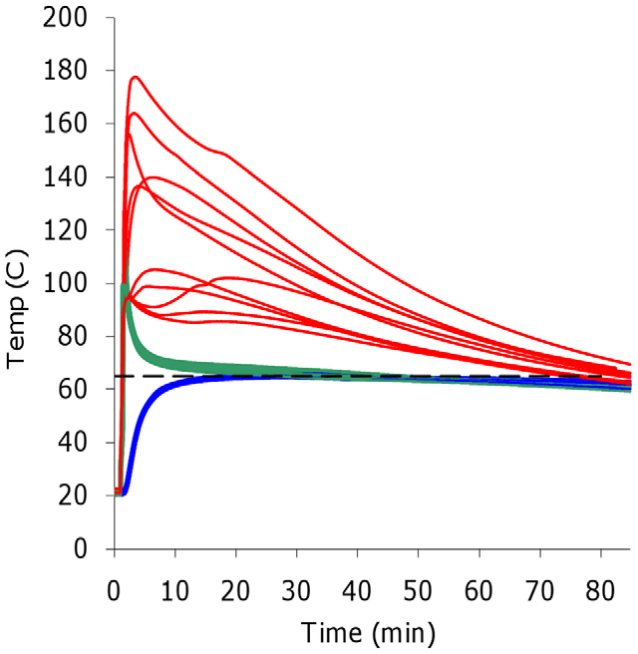
Temperature monitoring of the prototype designed for ∼65°C LAMP assays. Note the repeatability of results at three different locations over 10 replicate runs. (---)  =  target temperature (63°C). Red  =  Temperature of the CaO, Green =  Temperature at the CaO/EPCM interface, Blue =  Temperature of the amplification reaction. Sampling frequency  = 1 Hz.

Ten replicate runs of the optimized prototype displayed minimal variation in temperature from run to run within the reaction tubes (Figure 1). The heater reached the optimal incubation temperature in 15 minutes, and maintained the target temperature with minimal drift over 60 minutes. (Drift from minimum to maximum temperature within run, mean over all runs  = 2°C.) Comparison of the temperature plots for the CaO, EPCM, and reaction tubes in Figure 1 to Figure 1B in Hatano et al.[27] illustrates the beneficial effect of having the EPCM component in the heater. The CaO temperature traces show rapid and poorly controlled heat generation, with maximum temperatures exceeding 100°C. The traces of the EPCM at the interface with the CaO have a pattern similar to the CaO, but the initial temperature excursions are reduced in magnitude, and the plots are far more repeatable. Finally, the reaction tubes display only a uniform ramping to the target temperature followed by a prolonged stable isothermal phase. The temperature in the NINA reaction appears more uniform than that shown by Hatano et al.[27] for their hand-warmer device.

These results evince the potential of EPCM in an optimized design for controlling exothermic reactions in a simple NINA. This level of temperature control is important to enable conformance to the “sensitive”, “specific”, and “robust” aspects of the ASSURED guidelines. Once the abundant heat from the CaO reaction begins to melt the EPCM, the additional heat produced by the exothermic reaction is converted into the latent heat of fusion of the EPCM. Thus, the temperature in the EPCM remains constant at the selected melting temperature until the solid to liquid transition is complete (provided heat transfer within the EPCM is rapid). Once the CaO reaction has reached equilibrium, the energy stored as latent heat keeps the two-phase EPCM at the target temperature until complete solidification.

In our optimization work we observed that the purity of the CaO need not be high, although it should be consistent to yield consistent heat profiles (data not shown). The ability to use less pure CaO is important for minimizing the cost per amplification, addressing the “affordable” aspect of the ASSURED guidelines. Other key physicochemical parameters of raw CaO (particle size, particle porosity, presence of unoxidized calcium carbonate “grit”, etc.) that result from variation in kiln calcination of limestone[29] (the industrial manufacturing process) also must be kept consistent for consistent heat profiles. However, we were able to produce precise heat profiles in our prototypes with commodity grades of CaO. This makes the only disposable materials (CaO, water, and PCR tubes) in the device very inexpensive. The reaction of CaO and water can be tuned somewhat to control the steepness of the temperature ramp and the maximum temperature for a given reaction chamber, although flexibility and precision is greatly improved by including the EPCM.

The EPCM used here is tunable for many of its important characteristics (melting temperature range, specific heat capacity, thermal conductivity, etc.) making this device a flexible incubation platform potentially applicable to a number of isothermal amplification techniques. When evaluated by differential scanning calorimetry, the EPCM melts over a range of temperatures around the target (±2°C), and displays some hysteresis in the phase change, presumably due to polydispersity in polymer chain length and supercooling of the EPCM (personal communication from Renewable Alternatives). It is unclear at this time how this behavior contributes to variation seen in the results of the LAMP assay; however, the manufacturer of the EPCM is confident that further development of the EPCM for this application will mitigate this behavior. The EPCM is a fully hydrogenated fat product, so it is resistant to environmental oxidation and should be very stable. While the EPCM is not currently as readily available as CaO, and is not a commodity product like CaO, similar materials have been used in consumer products in the US. These EPCMs are made mainly from bio-based fats — namely beef tallow, palm oil, coconut oil and soybean oil — so local, low-cost production of the EPCM in the developing world should be feasible.

Portable energy for heat production could, of course, be supplied with conventional batteries, so a comparison seems appropriate. A cost analysis indicates that on a per calorie per test basis, using CaO as a thermal battery is several times less expensive than mass-produced, disposable, dry-cell batteries. Costs are scaled by the projected number of analytical runs possible and include both energy source and control hardware. CaO disposables are single use, while dry cells are expected to last five runs based on their energy density (four D-cells would be required). Two grades of CaO (reagent grade and soap grade), with an EPCM are compared to three possible dry-cell implementations (with an EPCM, with microprocessor closed-loop control, with thermostat closed-loop control). With a projected cost per run of US$0.56, the soap-grade CaO/EPCM combination is clearly the least-expensive alternative (compared to $1.40, $1.17, $1.21, and $1.16 for reagent-grade CaO/EPCM, D-cell/EPCM, D-cell/microprocessor, and D-cell/thermostat, respectively.) Costs were estimated from MSRP. Increased value of CaO over the alternatives could be realized at increased production volumes. Any special disposal or recycling required does not seem any more onerous than what is required for common batteries.

The data shown here were not gathered under any stringent external environmental control; therefore, given that testing was performed in an air-conditioned laboratory, it is likely that the system was not challenged in the same way as it would be at its intended point of use. The wide external temperature ranges found in LRS could significantly change the ramp time and/or duration at the desired temperature of the heater, possibly significantly, but the characteristics of the EPCM will ensure that the desired temperature is held for some period of time, regardless – *without calibration to the ambient conditions or closed-loop control*. First principles of heat transfer dictate that the effects of ambient on ramp time and/or duration should be greatest when the desired temperature is furthest from ambient. Thus, the problem should be appropriately non-dimensionalized to identify states of similitude. We plan to explore these phenomena and to evaluate their effects once we have improved our understanding of the intrinsic variation in the assay chemistry sufficiently to evaluate those effects. This evaluation will include trials under actual field conditions.

### LAMP Assay Demonstration and Comparison to a Reference Heater

**Figure 2:**
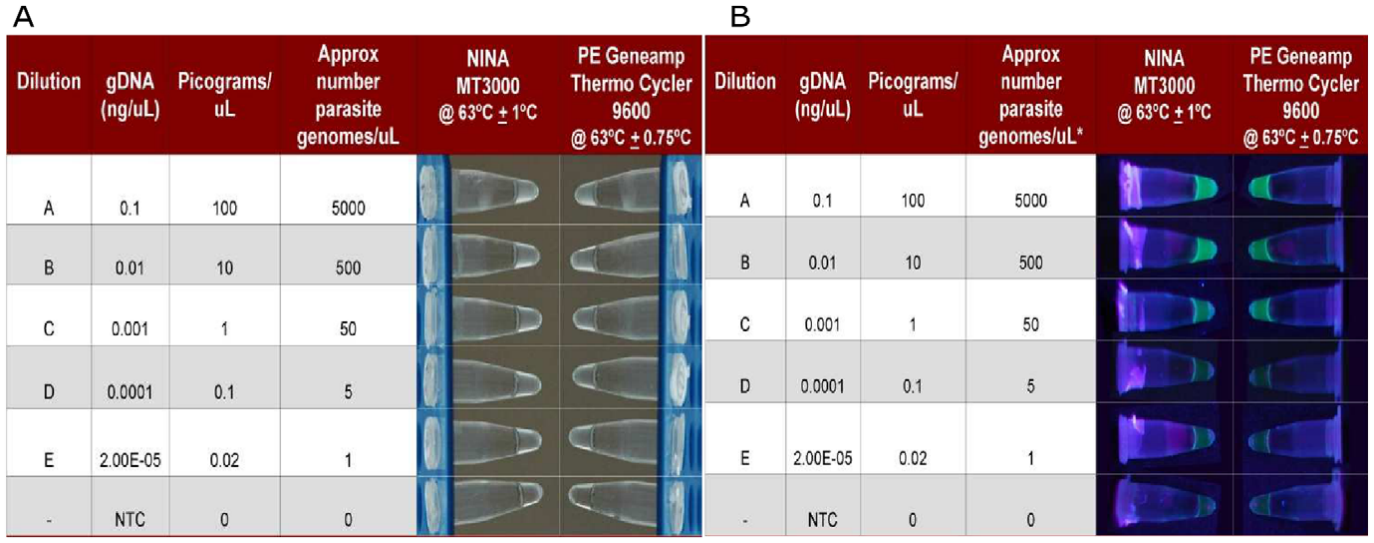
LAMP with qualitative visual readouts performed in both the NINA heater and a reference instrument. LAMP assays were performed for a dilution series of *P. falciparum* genomic DNA (see figure for concentrations) with qualitative visual readouts and with amplification performed in both the NINA heater and a reference instrument (Perkin Elmer GeneAmp Thermocycler 9600, set at 63°C). NTC =  no template control. A) A turbidimetric readout based on the scattering of accumulated magnesium pyrophosphate precipitate (a by-product of the amplification reaction).[37] B) A fluorescence readout based on the Loopamp Calcein reagent. Pyrophosphate (a by-product of the amplification reaction) competitively displaces the Calcein fluorophore from manganous ions (Mn++), relieving the quenching effect of the Mn++. Further fluorescent enhancement results from the binding of magnesium ions (Mg++) by Calcein at the site vacated by Mn++.[30]

Representative images of the qualitative results (Figure 2) shows 1) the NINA heater is capable of supporting LAMP, 2) that samples incubated in the NINA heater give results that are virtually identical to those incubated in parallel in the GeneAmp® 9600. For both incubators the turbidimetric readout method (Figure 2A) is difficult to interpret, but turbidity due to accumulating LAMP product is observed (relative to the no-template control, or NTC). The fluorescence of the Calcein reagent when illuminated with a UV lamp (Figure 2B) is more easily seen as an increase in intensity (relative to NTC) for the dilutions that are >1 pg/µL. Note that there is some background fluorescence visible in the NTCs with *both* heaters. These observations conform to those noted by the operator at the actual time of the analysis, so no artifacts have been introduced by the photographic process.

**Figure 3:**
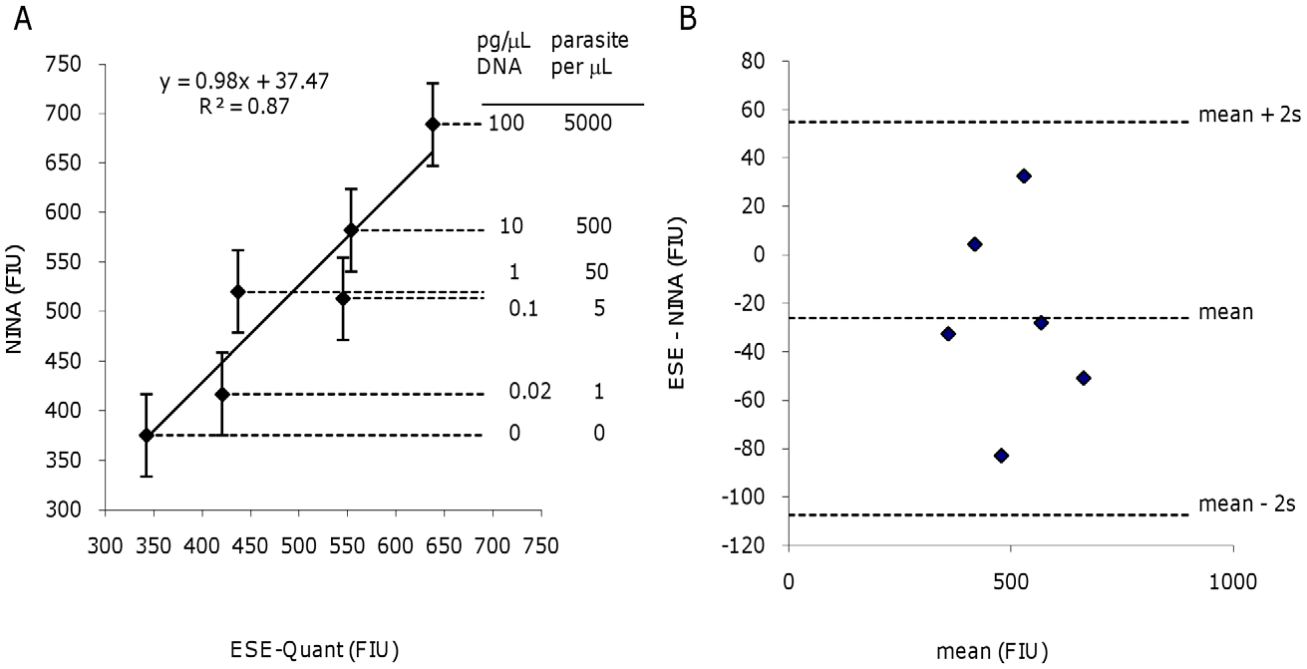
Quantitative method comparison of LAMP performed in both the NINA heater and a reference instrument. LAMP assays were performed for a dilution series of *P. falciparum* genomic DNA (see figure for concentrations), with amplification performed in both the NINA heater and a reference instrument (ESE-Quant Tube Scanner, set at 63°C) for the same amount of time. Fluorescence intensity of the Calcein dye was then read on the SpectraMax M2 plate reader with λ_ex_ = 485 nm and λ_em_ = 515 nm. A) Linear regression analysis of the method comparison. The error bars represent ±2 s using the best unbiased estimate for replicate noise available from the data set. B) Bland-Altman analysis of the same data.

A quantitative comparison of Calcein fluorescence corroborates the qualitative study. A statistical method comparison by the two most common techniques indicates substantial quantitative agreement between samples incubated in the NINA heater to those incubated in parallel in the ESE-Quant Tube Scanner. Linear regression of the fluorescence intensity units (FIU) observed for samples incubated in the NINA heater as a dependant variable of the FIU observed for samples incubated in the ESE-Quant (Figure 3A) results in a slope of 0.98 and a y-intercept of 37.5 FIU, with a coefficient of determination of 0.87. Bland-Altman analysis (Figure 3B) reveals a mean difference (ESE – NINA) of −26 FIU, no dependence of difference on mean, and all differences lie within the ±2s interval that indicates the differences are random, not systematic. Although these experiments were intended to quickly assess the agreement between heater types and were not designed to rigorously define the dose response relationship of a nascent assay, closer inspection of the FIU for each concentration (Figure 3A) reveals a general increase in response with increasing dose, within the experimental noise limits of this admittedly small sample set. As with the qualitative assay demonstration, considerable background fluorescence was observed in NTC reactions in *both* heaters (Figure 3A and 3B).

These results clearly show that the NINA heater can incubate isothermal reactions predictably and precisely with no electricity and without any form of closed-loop control. We also demonstrate that it can be used for LAMP assays, with no discernable difference when compared to two reference heaters, the GeneAmp® 9600 and the ESE-Quant Tube Scanner. There is a bias between the NINA heater and the ESE-Quant (NINA higher), but this is not a significant finding considering we are comparing FIU without any assay calibration. This bias would be easily removed by applying a standard curve. Although we did not intend to rigorously qualify the LAMP assay for malaria here, these results suggest that a quantitative assay with a clinically significant lower limit of detection and three decade dynamic range might be possible with further development of the protocol. Planned work will comprehensively compare incubation of several isothermal assays with the NINA heater to incubation with conventional, electrically-powered instruments by many metrics – sensitivity, specificity, accuracy, precision, and other standard figures of merit must all be assessed before equivalence can be rigorously inferred. However, these prelimary results are very encouraging.

### Other Isothermal Techniques

**Figure 4:**
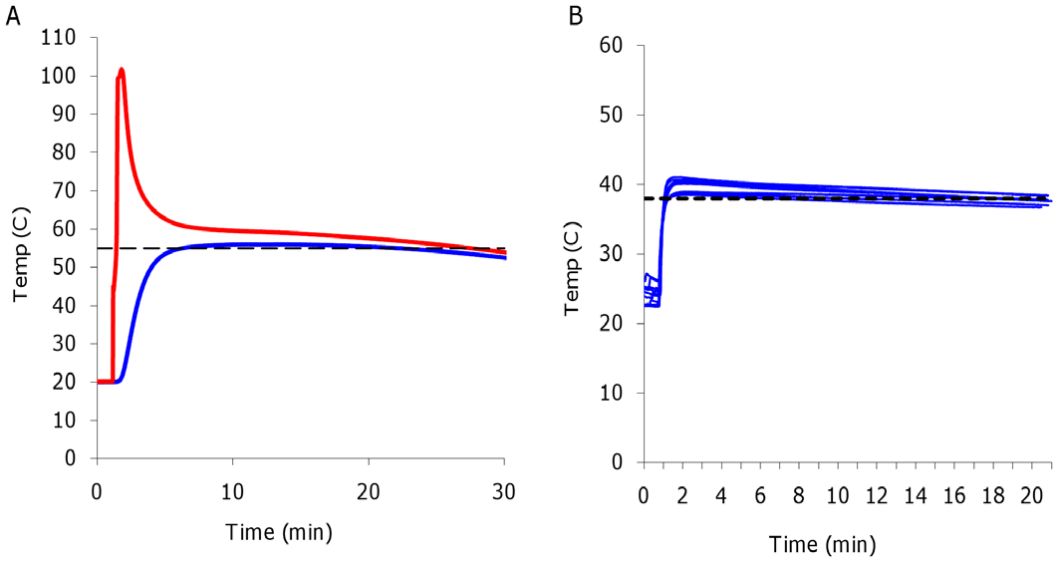
Temperature monitoring of prototypes designed for other contexts. A) A representative plot for a CaO prototype with a temperature set point of 55°C, suitable for EXPAR assays. B) Ten replicate plots of the NaAc prototype with a temperature set point of 38°C, suitable for a variety of uses. As yet, this prototype has not been exhaustively optimized for precision as have the CaO/EPCM based units. Red =  Temperature of the CaO, Blue =  Temperature of the amplification reaction. ( --- )  =  target temperature. Sampling frequency = 1 Hz.

We have also explored heaters with temperature profiles suitable for other isothermal amplification techniques requiring different incubation temperatures, e.g., the Exponential Amplification Reaction (EXPAR), Nicking Enzyme Amplification Reaction (NEAR), or Recombinase Polymerase Amplification (RPA), could be integrated with this method. These prototypes are not significantly different in form, but use different EPCMs, and in one case a different exotherm. A CaO heater with a different EPCM formulation has been shown to yield a temperature profile suitable for EXPAR with a nominal temperature of 55°C (Figure 4A). Evaluation with EXPAR reactions are in process. We have also explored a similar heater approach with sodium acetate (NaAc, Figure 4B). Hand warmers based on the crystallization reaction of NaAc are common. In a purified form, at typical ambient temperatures, liquid NaAc is thermodynamically unstable but kinetically stable due to the absence of nucleating sites for crystal formation. The application of a mechanical shock initiates the exothermic crystallization, and when mixed as a 25% aqueous solution the phase change occurs repeatably at ∼37°C. In this system, NaAc acts as both the exothermic reactant and the EPCM. This system has the advantage of being regenerable (immersion of the NaAc in boiling water is sufficient). For isothermal amplification methods operating at temperatures below 45°C as well as for other diagnostic applications requiring heating (e.g., smart-polymer-based analyte pre-concentration[30]), NaAc is the preferred exothermic/phase change system. These results establish that the heater is a flexible platform for a number of isothermal detection techniques.

### Assay Specific Limitations of This Investigation

We have shown results for an instrument-free LAMP assay with a simple qualitative visual readout. As operated here, LAMP is an exponential rate assay being assessed with an endpoint measurement. Thus, the timing of reaction interrogation and/or a reliable “stop” reaction are required for quantitative precision. If quantitative results are required, improvements to the entire assay system to facilitate precise timing will be necessary. This could include, for example, a different heater-lid or incubation-vessels to facilitate access, or a “reading window” in the heater to enable visual interrogation while the vessel is still in the heater. An elevated temperature “stop” (>80°C) is generally used for LAMP. In our experimental work here, we used an electrical heat block for this purpose; however, this could be accomplished with the electricity-free heater by the inclusion of a parallel heating unit at a higher temperature (essentially, by including a second incubation chamber that uses a different EPCM, or no EPCM at all). Alternatively, a chemical “stop” could be developed, or a boiling water bath could be kept at hand. Other assays perform best with a pre-amplification, high-temperature denaturation step (“hot start”). A second incubator chamber could facilitate this feature even more readily.

These data were gathered on contrived samples diluted in buffer. It has already been demonstrated that LAMP assays can be performed on clinically relevant specimens without NA extraction/purification and without a pre-amplification, high-temperature denaturation step.[31]–[35] Recent results of an HIV assay on the NINA platform with clinical samples from HIV-positive infants will be reported elsewhere.

Furthermore, neither turbidity nor Calcein reactions are sequence-specific signals—as a result non-specific amplification will also produce a strong signal—a possible cause of the NTC background fluorescence noted above. Greater analyte specificity should be possible by incorporating a fluorescent molecular beacon probe specific to an internal region of the target amplicon, thus minimizing non-specific signal. [28] Alternatively, the amplified product could be the input to a lateral flow strip test with a visual readout. [36]–[39]

Any of these potential improvements should be approached with a secondary aim of minimizing the potential for amplicon contamination from previous tests. Wherever possible, opening of the amplicon container after amplification should be avoided. This may be challenging if molecular beacon quenchers need to be added, or aliquots for ICS testing need to be removed. Regardless of how the system and assays are improved, we have clearly demonstrated that the NINA heater is an effective device that can facilitate the electricity-free amplification of NA using an isothermal technique.

### Future Directions

There are several applications of this technology that could have an impact on diagnostics for LRS. One application is as a modular amplification unit where a sample and the required reagents would be introduced to the heater and amplified product withdrawn for subsequent analysis by any simple detector. In this embodiment a standard PCR tube can be the reaction chamber and could be used later as a cuvette for fluorometric analysis to resolve the presence of amplicons. This would free the user from the high power requirements of electrical heating but would still require some sort of detection instrument or device with its attendant requirements. One could also imagine how a properly tuned, stand-alone heater unit could be applicable to any field analytical or preparative method that requires a constant heat source; e.g., cell lysis or temperature-responsive polymer mediated concentration. More compelling is the potential of the NINA heater as the core component of a stand-alone assay kit, capable of providing a result without external electrical power, a reader instrument, or any complex ancillaries. Such a device might include the NINA heater, reaction chambers containing lyophilized reagents, sample metering devices, a readout chamber or lateral flow strip for visible interrogation, and an LED “penlight” for fluorescence excitation (if required). We envisage versions of the kit that are fully disposable (for high-value applications in developed countries such as for home testing and for first-responder biothreat detection) and partially reusable (primarily for LRS use). Most of the components necessary to create such a NINA kit already exist. We are currently working to combine them into a field-ready, instrument- and electricity-free, sample-to-result, molecular diagnostic test system (Figure S1).

### Conclusion

We have demonstrated the ability of an optimized NINA heater prototype, based on exothermic chemical reactions and EPCM, to support isothermal NA amplification assays and established its equivalence to commercially available PCR instruments. The disposable heater described is a component of an instrument-free point of care molecular diagnostics system under development. When combined with other innovations in development that eliminate power requirements for sample preparation, cold reagent storage, and readout, the NINA heater will comprise part of a kit that enables electricity-free NA testing for many important analytes. Replicate temperature profiles display minimal variation between runs and far less variation than any similar devices, highlighting the advantages of including an EPCM in the design. Versions of the prototype for several isothermal techniques have been presented, clearly evincing the potential of the NINA heater.

## Materials and Methods

### Materials

**Figure 5:**
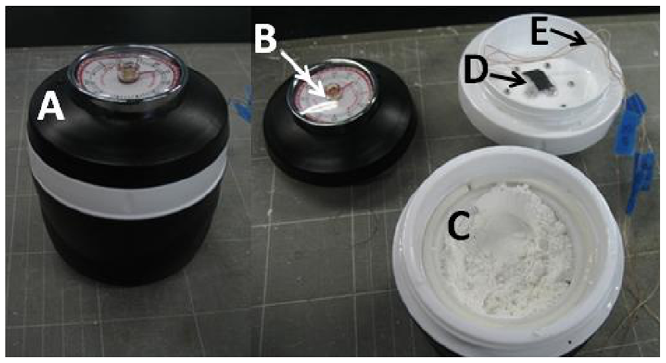
Fifth-generation prototype design made from a reusable $4 insulated soup thermos container. (A) assembled incubator, (B) incubator lid with built-in spring timer, (C) CaO chamber (w/ CaO added), (D) assay tubes, and (E) thermocouple wires (only required in temperature monitoring experiments).

In the NINA heater for LAMP, we used the exothermic reaction of calcium oxide (CaO, or quicklime; Science Stuff, Inc., Austin, TX, USA, Cat # C1450) and water to generate the necessary heat. To keep the isothermal device within the temperature band required for LAMP, the reaction chambers were surrounded with an engineered fat-based compound with a high specific heat capacity and specific melting range centered around 65°C (Renewable Alternatives, Inc., Columbia, MO, USA). While several prototype heater designs have been explored, the optimized heater uses an off-the-shelf insulated food storage container (a “thermos”) to provide an insulated housing with two chambers (Figure 5). The bottom chamber contains the exothermic reaction, and the upper chamber contains the EPCM and reaction wells. To facilitate directed heat transfer to the reaction wells, an aluminum “honeycomb” material (Plascore, Inc., Zeeland, MI, USA) was added to the upper chamber prior to introduction of the EPCM. The machined reaction wells, sized to closely fit a standard 200-µL PCR tube, are embedded in the EPCM. Three reaction wells were used for most prototypes (one for a positive control, one for a negative control, and one for an unknown specimen); however, the existing prototype could easily be modified to accept several times this number without significant loss of performance, based on the available space in the EPCM and first principles of heat transfer. An inexpensive spring timer (manufacturer's suggested retail price [MSRP] ≈10 US$) with an audible “ready” indicator was affixed to the lid of the heater unit for added electricity-free functionality.

Loopamp® DNA LAMP kits were purchased from Eiken Chemical Co. Ltd (Tokyo, Japan, Code No.: LMP206). The Eiken Loopamp® Fluorescence Detection Reagent (Code No.: LMP221), a Calcein-based reagent that indirectly indicates the progress of DNA amplification in the LAMP reaction, was used for fluorescence experiments. LAMP primer sequences for *P. falciparum* (Integrated DNA Technologies , Coralville, IA, USA) were as described by Poon et al.[40] Genomic DNA from *P. falciparum* for preparing contrived samples was obtained from the PATH laboratory specimen collection.

Either an ESE-Quant Tube Scanner (ESE GmbH, Stockach, Germany) or a PE GeneAmp® Thermocycler 9600 (Applied Biosystems, Carlsbad, CA, USA ) was used as a quantitative reference instrument for temperature incubation. The ESE-Quant Tube Scanner also served as a reference for quantitative fluorescence measurement. A SpectraMax M2 fluorescence plate reader (Molecular Devices, Sunnyvale, CA, USA) was also used (as noted in individual experiments). Physitemp IT-23, T-type thermocouples (Clifton, NJ, USA) and an Omega DaqPRO 5300 Data Recorder (Omega Engineering, Stamford, CT, USA) were used to monitor temperature.

### Methods

Thermocouples were installed in a reaction well (below its microcentrifuge tube) at the bottom of the chamber containing the EPCM and in the exothermic reaction chamber for experiments performed to characterize the temperature profiles of the heaters. The temperature acquisition rate was 1 Hz. Initial experiments were focused on refining the dimensions of the heater, optimizing the quantity and quality of CaO and water, and testing EPCM formulations—with the goal of minimizing initial pre-heating time and variability during the specified incubation period. In the optimized device, 20 gm of CaO and 6.8 mL of water were added to the bottom chamber and mixed by rotary stirring for five strokes to initiate the heating and then the components were assembled as discussed above.

To verify that the device could incubate a LAMP assay, the Eiken kits were used as per package insert instructions, except where noted below. Clinically relevant dilutions of genomic DNA were made in Eiken kit buffer to yield the DNA concentration and approximate parasite count noted for each experiment. All dilutions were prepared as single solutions and then aliquoted across treatment conditions to minimize preparation variation. No template controls (NTC) were prepared first and immediately sealed to reduce the possibility of contamination. Mineral oil was layered on the tops of the samples to minimize evaporation. All reactions were incubated at 63°C.

Qualitative readout experiments were performed both with and without the Calcein reagent to determine if turbidimetric readouts were possible. To compare the performance of the NINA heater to a reference heater, these experiments were also performed in parallel with reactions incubated in both the test device and in the GeneAmp® thermocycler, programmed for a constant incubation at 63°C.

Quantitative fluorescence experiments were performed in parallel with reactions incubated in both the test device and in the ESE-Quant Tube Scanner, programmed for a constant incubation at 63°C. For NINA incubated reactions, LAMP was terminated at the time (∼36 min.) when the signal from the parallel reactions on the Tube Scanner began to indicate detectable amplification to avoid signal saturation in all dilutions. Termination was accomplished by flash chilling and later inactivating the reaction by heating at 80°C for 5 minutes. The fluorescence signals of the NINA incubated samples were then read on the SpectraMax M2 plate reader with λ_ex_ = 485 nm and λ_em_ = 515 nm.

## Supporting Information

Figure S1 **The workflow of a proposed NA amplification assay kit.** The kit will be an instrument-free, electricity-free nucleic-acid amplification test that is compatible with whole blood, is temperature stable and contains contamination. 1) Initiate NINA heater by installing heater cartridge (a) into insulated housing (b), add EPCM module (c) and lid (d). 2) Set up for assay by opening single assay subkit. 3) Sample blood to calibrated line on collection capillary. 4) Transfer blood and blister contents to “S” tube and prefilled diluent to “NC” and “PC” tubes and mix all. 5) Amplify. Verify temperature “ready” indication on the NINA device through transparent view port in the lid, remove the lid, add the three tubes to the NINA heater, and replace lid. Incubate 45 minutes. Verify temperature is still in range through transparent view port (process control). 6) Quench to all three tubes by pushing cap to burst frangible seal and transfer ∼10 µL diluted quencher to the amplified mixture.
(TIF)

Table S1 **Table of references to posters and patents about the NINA exothermic-heat/EPCM heaters.**
(PDF)
