# Supplementary figures and images for: A Simple, Inexpensive Device for Nucleic Acid Amplification without Electricity—Toward Instrument-Free Molecular Diagnostics in Low-Resource Settings
Source: PLoS One. 2011 May 9;6(5):e19738. doi: 10.1371/journal.pone.0019738 (PMC3090398; doi:10.1371/journal.pone.0019738)

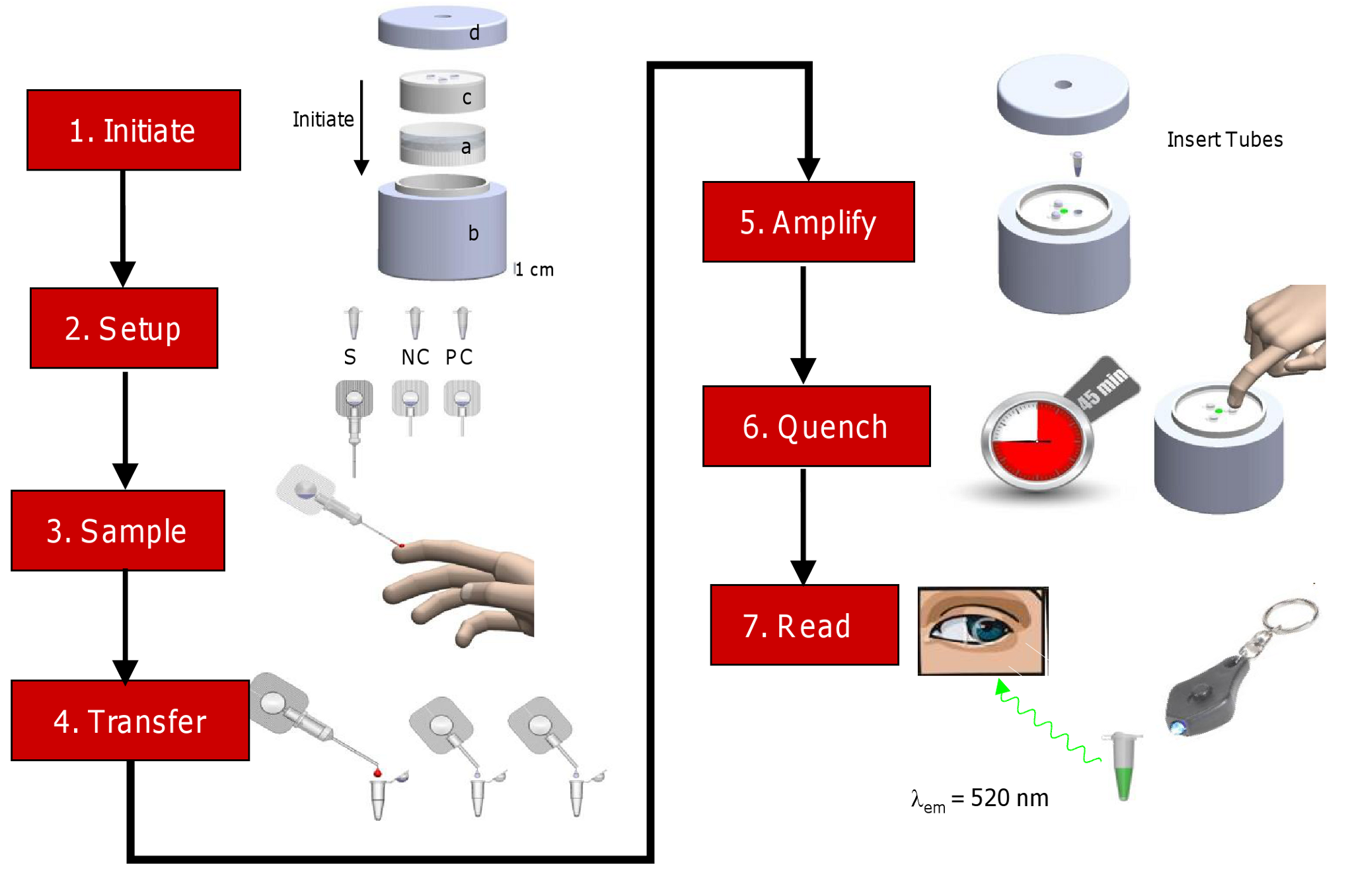

Supplement: Figure S1 — The workflow of a proposed NA amplification assay kit. The kit will be an instrument-free, electricity-free nucleic-acid amplification test that is compatible with whole blood, is temperature stable and contains contamination. 1) Initiate NINA heater by installing heater cartridge (a) into insulated housing (b), add EPCM module (c) and lid (d). 2) Set up for assay by opening single assay subkit. 3) Sample blood to calibrated line on collection capillary. 4) Transfer blood and blister contents to “S” tube and prefilled diluent to “NC” and “PC” tubes and mix all. 5) Amplify. Verify temperature “ready” indication on the NINA device through transparent view port in the lid, remove the lid, add the three tubes to the NINA heater, and replace lid. Incubate 45 minutes. Verify temperature is still in range through transparent view port (process control). 6) Quench to all three tubes by pushing cap to burst frangible seal and transfer ∼10 µL diluted quencher to the amplified mixture. (TIF) [file pone.0019738.s001.tif]
