# Supplementary material for: A Simple, Inexpensive Device for Nucleic Acid Amplification without Electricity—Toward Instrument-Free Molecular Diagnostics in Low-Resource Settings
Source: PLoS One. 2011 May 9;6(5):e19738. doi: 10.1371/journal.pone.0019738 (PMC3090398; doi:10.1371/journal.pone.0019738)
Supplement: Table S1 — Table of references to posters and patents about the NINA exothermic-heat/EPCM heaters. (PDF) [file pone.0019738.s002.pdf]

1. USPTO application #20090036665
2. USPTO application # 20090004732
3. LaBarre P, Gerlach J, Wilmoth J, Beddoe A, Singleton JL, Weigl BH. *LAMP without electric heat: A chemically-heated, non-instrumented nucleic-acid amplification assay platform for point-of-care use* [poster]. Presented at: AACC 41st Annual Oak Ridge Conference, April 16, 2009; Baltimore, MD
4. LaBarre P, Gerlach J, Wilmoth J, Beddoe A, Singleton JL, Weigl BH., *HOT Diagnostic Technologies: Low-cost, point-of-care nucleic acid amplification using chemical heat to replace traditional heat sources* [poster]. Presented at: 6th Annual World Health Care Congress, April 12, 2009; Washington, DC; also, same authors, *Non-Instrumented Nucleic Acid Amplification (NINA): Instrument-Free Molecular Malaria Diagnostics for Low-Resource Settings* [invited talk]. 32nd Annual International Conference of the IEEE EMBS, August 31 - September 4, 2010, Buenos Aires, Argentina)
